# Complete mitochondrial genome of a treefrog, *Hyla sp.* (Anura: Hylidae)

**DOI:** 10.1080/23802359.2017.1310606

**Published:** 2017-04-12

**Authors:** Dong-Ha Nam, Hyun-Ah Lee, Eun-Bin Kim, Geun-Joong Kim, Eung-Sam Kim, Chungoo Park, Ha-Cheol Sung, Dong-Hyun Lee

**Affiliations:** a Department of Biological Sciences, College of Natural Sciences, Chonnam National University, Gwangju, Korea;; b School of Biological Sciences and Technology, College of Natural Sciences, Chonnam National University, Gwangju, Korea

**Keywords:** *Hyla sp*., Hylidae, mitochondrial genome

## Abstract

The complete mitochondrial genome of a treefrog (*Hyla sp.*) was determined. The circular mitochondrial genome is 18,288 bp long and encodes 13 proteins, 22 transfer RNAs, and 2 ribosomal RNAs. Phylogenetic analysis of its full genome sequences showed that *H. sp.* was closely related to *H. ussuriensis* and *H. japonica* rather than *Dryophytes suweonensis*, consistent with the results from each protein-coding gene and a cluster between 12S rRNA and 16S rRNA. The present study will provide essential genomic information for biogeographical distribution and evolutionary history of an endemic treefrog, *H.sp.*

The genus *Hyla* distributed widely in North America, Eurasia, and Africa, with more than 35 species (Li et al. 2015). Of these, two treefrog species such as *Hyla japonica* and *H. suweonensis* have been identified in Korea. Unlike *H. japonica* with a widespread distribution throughout North East Asia, the *H. suweonensis* is endemic to Korea, with a restricted distribution around the western coastal plains of Korea. Systematics and biogeography of the genus *Hyla* on the East Asian species indicates that *H. japonica* and *H. suweonensis* are potentially synonymous to *H. ussuriensis* and *H*. *immaculate* from China, respectively (Li et al. 2015). Divergence-time estimation, diversification and ancestral-area reconstruction analyses also suggest that genus *Hyla* can be changed to *Dryophytes* (Li et al. 2015; Duellman et al. 2016). However, the complete mitochondrial genome of *D. suweonensis* (as the synonym of an endangered treefrog, *H. suweonensis*) was not generated until recently (Borzée et al. 2017), and its genetic information on the geographical distribution is unfortunately limited. Here, we sequenced the complete mitochondrial genomes of an endemic treefrog, *H. sp.* by next-generation sequencing (NGS) technology, which can be helpful for its evolutionary and phylogenetic analyses.

The specimens of *H. sp.* for buccal swabbing were collected from Iksan (35.950° N; 126.958° E), Korea, and the total genomic DNA was extracted from an adult male (stored at the Wildlife Specimen Bank in Chonnam National University, Korea), considering initially as a *H. suweonensis* based on its vocal characteristics, habitat selection, and morphological properties (Ham 2014). Following quality control and library construction of the specimen, the NGS reads (450-bp length in each read) generated from MiSeq (Macrogen, Seoul, Korea). Approximately 0.25% mapped reads (71,863 out of 28,476,666) were used for *de novo* assembly and annotation by using commercial software (MITOS) to identify a complete mitochondrial genome with about an average 150 × coverage.

The complete mitochondrial genome of *H. sp.* was 18,288 bp in length, which contains 37 genes for 13 proteins, 22 transfer RNAs (tRNAs), 2 ribosomal RNAs (rRNAs), an origin of light strand replication site (O_L_), and a long noncoding region called the control or D-loop region with a 202bp gap (GenBank: KY700829). The overall nucleotide composition for *H. sp.* was 30.1% A, 25.5% C, 14.3% G, and 30.1% T. The phylogenetic analysis was performed using MEGA6 to construct a neighbour-joining tree with 1000 bootstrap replicates containing complete mitochondrial genomes of 13 species derived from sister-genera *Hyla* and *Dryophytes* (Figure 1). *Bombina orientalis* was used as an outgroup for tree rooting. The phylogenetic tree indicated that *H. sp.* showed closer evolutionary distances with *H. ussuriensis* compared with *D. suweonensis* (Borzée et al. 2017). Variants from the full genome sequences of *H. sp.* were approximately 10% when compared to that of *D. suweonensis,* although the specimen used in the present study was considered initially to *D. suweonensis* based on its morphological and vocal features (Ham 2014). The variation in amino acid sequences from 13 protein coding genes was an average of 0.90% (range: 0–2.65%) between *H. sp.* and *H. japonica,* and 3.06% (0.19–5.67%) between *H. sp.* and *D. suweonensis*. The phylogenetic relationships based on amino acid sequences of each protein-coding gene and a cluster between *12S rRNA* and *16S rRNA* (nucleotides of 12S rRNA – tRNA^Val^ – 16S rRNA) also supports the aforementioned phylogenetic relationships among the genera *Hyla* and *Dryophytes* species (Figure 1).

**Figure 1.**
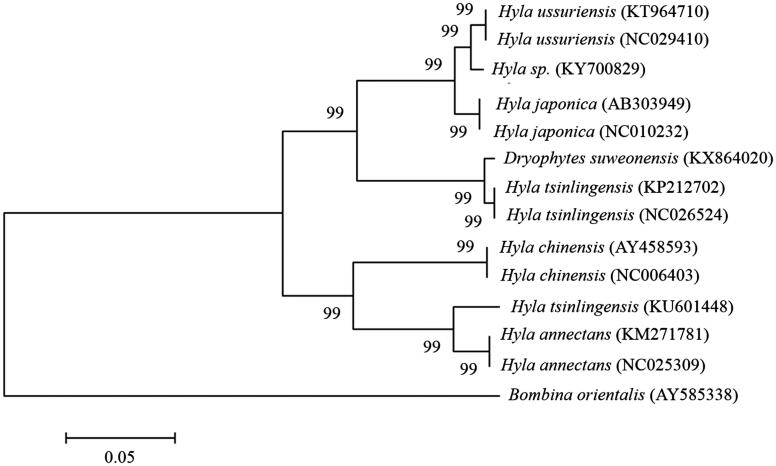
Phylogeny of *H. sp* and other related species based on complete mitochondrial genome sequences. The complete mitochondrial genomes were downloaded from GenBank and the phylogenetic tree is constructed by a neighbour-joining method with 1000 bootstrap replicates containing the full genomes of 13 species derived from *Hyla* and *Dryophytes*. *B. orientalis* was used as an outgroup for tree rooting. GenBank accession numbers of each mitochondrial genome sequence are given in the bracket after the species name.

The complete mitochondrial genome sequences of *H. sp.* may provide intriguing insight into biogeographical distribution and conservation genetics of this endemic species, and to evoke to do further researches to reveal the behavioural and morphological relationships among *Hyla* and *Dryophytes* species.
